# NXP-2-positive dermatomyositis presenting with pronounced unilateral lower extremity subcutaneous edema

**DOI:** 10.1016/j.jdcr.2025.06.018

**Published:** 2025-06-24

**Authors:** Madisen A. Swallow, Alana Deutsch, Amanda Zhou, Jeff R. Gehlhausen, Matthew Lempel, David Fernandez, Lana Bernstein, Jean Bolognia, Sarika M. Ramachandran

**Affiliations:** a Yale School of Medicine, New Haven, Connecticut; b Department of Dermatology, Yale School of Medicine, New Haven, Connecticut; c Department of Rheumatology, Yale School of Medicine, New Haven, Connecticut; d Department of Rheumatology, Hospital of Special Surgery, New York, New York; e Rheumatology Section Head, Greenwich Hospital YNHH, Greenwich, Connecticut

**Keywords:** dermatomyositis, edema, leg, unilateral

## Introduction

In dermatomyositis, there are characteristic cutaneous findings, for example Gottron papules and cuticular telangiectasias, as well as the production of autoantibodies.^1^^,^^2^ Several of these autoantibodies are associated with distinctive clinical presentations. In patients with anti-nuclear matrix protein 2 (NXP2) antibodies, the classic cutaneous findings of dermatomyositis are often accompanied by symmetric peripheral edema, calcinosis cutis, dysphagia, and myositis in addition to an increased risk of an underlying neoplasm.^1^ We report a patient with anti-NXP2 antibody-positive dermatomyositis who presented with unilateral lower extremity edema.

## Case report

A healthy 56-year-old woman with no significant past medical history and on no chronic medications presented with a 6-week history of left lower extremity (LLE) erythema, warmth, and swelling, plus a progressive pruritic eruption, which the patient described as blotchy pink patches. Two days after the onset, she was exposed to narrowband ultraviolet B radiation via a home phototherapy unit utilized for photorejuvenation. She was diagnosed with cellulitis of the LLE by her local dermatologist and completed a 10-day course of doxycycline (100 mg twice daily). Due to a lack of improvement, she was admitted to an outside hospital with the diagnosis of refractory cellulitis and received 2 days of intravenous vancomycin, piperacillin/tazobactam, and ertapenem, followed by a 7-day course of oral cephalexin. A computerized tomography (CT) scan of the LLE noted panniculitis for which she was started on prednisone (1 mg/kg/day), tapered over 3 weeks. There was improvement of the LLE erythema and edema, but within days of completing the course of prednisone, both recurred. Her dermatologist performed biopsies of the left thigh and upper back, which were read as interface dermatitis consistent with erythema multiforme. Four days following the conclusion of her prednisone taper, she was referred to our facility for further evaluation and treatment.

**Fig 1:**
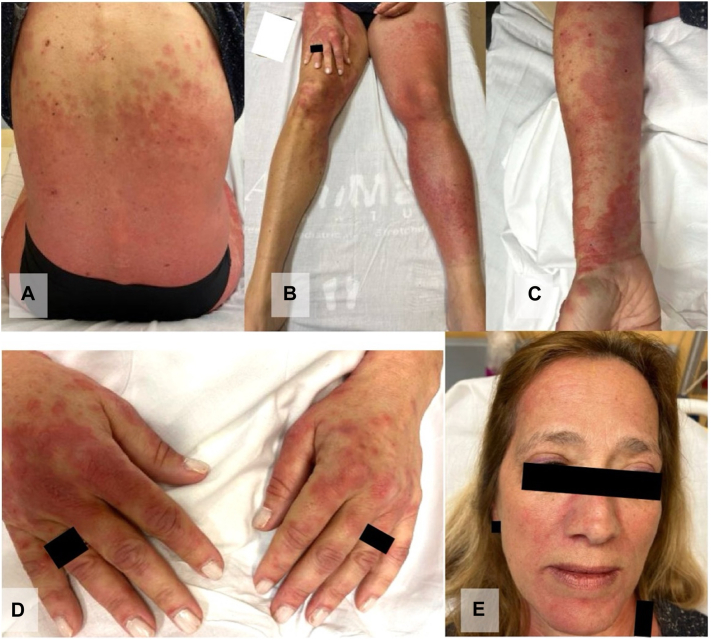
Representative clinical images from the time of presentation as shown. Coalescing, blanchable, pink-red macules and papules on the mid to lower back and upper thighs **(A)**; pink-red macules and papules on the right hand and both lower extremities with coalescence of lesions and marked swelling of the left lower extremity **(B)**; and dusky red papules and plaques on flexor forearm **(C)** and the dorsal hands extending onto the fingers **(D)**. On the face, there are pink-red plaques on the forehead, nose, mid to lower cheeks, with notable involvement of the nasolabial folds, superior portion of the upper cutaneous lip, and chin **(E)**.

On admission, she had coalescing, blanchable, pink-red macules and papules on her neck, chest (with a relative V-neck cutoff), abdomen, back, and upper thighs (Fig 1). There were symmetric, thin, pink-red plaques on the forehead, nose, cheeks, with notable involvement of the nasolabial folds, lip, and chin (Fig 1, *E*). Areas of sparing included skin above the eyebrows, lower eyelids, nasal bridge, and lower portion of the upper cutaneous lip. Superior to the margin of the upper eyelids were thin violaceous linear lesions accompanied by mild edema (Fig 1, *E*). On the dorsal hands and fingers were red papules and plaques, some with dusky centers and/or pseudovesiculation (Fig 1, *E*). Cuticular erythema was present, but neither dilated capillary loops nor avascular areas were seen by dermoscopy. Marked swelling of the LLE, from the inguinal fold to the foot, was observed (Fig 1, *B*). According to rheumatology’s initial examination, she had 3/5 strength in the proximal left lower extremity with full strength preserved in all other muscle groups of all extremities.

**Fig 2:**
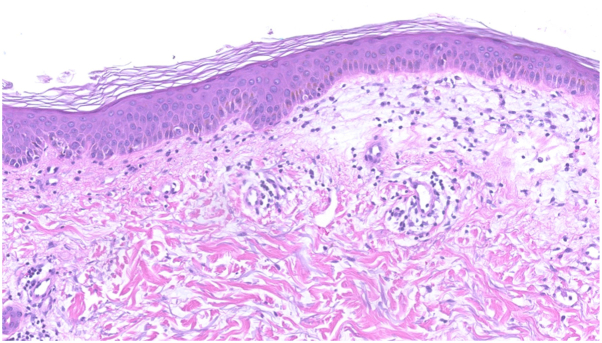
Photomicrograph of left thigh punch biopsy demonstrates papillary dermal edema, subtle vacuolar interface dermatitis, and a superficial and mid-dermal lymphocytic infiltrate with increased dermal mucin (100× original magnification).

Admission laboratory studies that were within normal limits included a complete blood count with differential, comprehensive metabolic panel, CRP, ESR, C3, C4, and CA125. The only abnormal value was hypoalbuminemia (2.6 g/dL). Serum creatine kinase and aldolase were 9× (1851 U/L; ref 11-204 U/L) and 3× (26.1 U/L; ref ≤8.1 U/L), the upper limits of normal, respectively. The ANA titer was <1:80, and anti-SSA and -SSB antibodies were 0.8 and 0.2 (ref: 0.0-0.9 AI). Myositis panel, which was performed using a radioimmunoprecipitation assay, detected anti-NXP2 antibodies. No deep vein thromboses were noted in either lower extremity by duplex ultrasound, while magnetic resonance imaging of the left thigh demonstrated edema throughout the subcutaneous plane, muscle, and deep intermuscular fascial planes. Punch biopsy of the left thigh revealed marked papillary dermal edema with subtle vacuolar interface dermatitis (Fig 2). In the dermis, there was a superficial and mid-dermal lymphocytic infiltrate with increased mucin.

**Fig 3:**
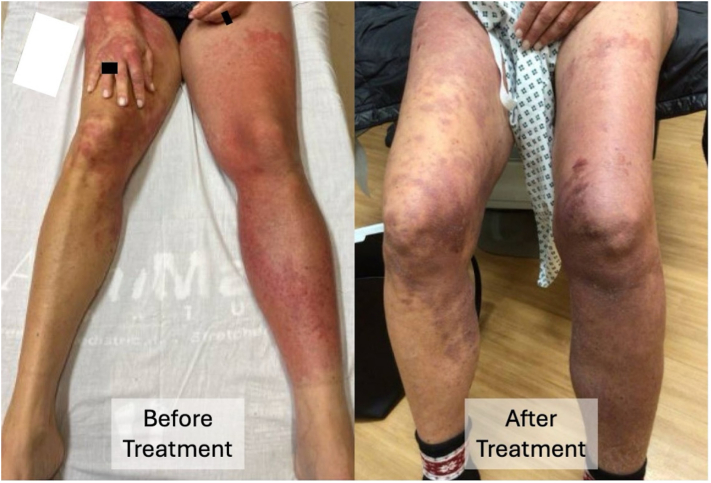
Clinical improvement in the erythema and edema of the left lower extremity following 1 month of treatment with prednisone and IVIG, plus the later addition of mycophenolate mofetil. IVIG: intravenous immunoglobulin.

She was diagnosed with NXP-2-positive dermatomyositis and treated with triamcinolone 0.1% cream, prednisone (1 mg/kg/d × 4 wk, followed by a planned taper of 10 mg weekly), and intravenous immunoglobulin (2 g/kg every 4 wk). Mycophenolate mofetil (1000 mg twice daily) was added after hospital discharge as she had noted weakness of her LLE, requiring her to ambulate with a cane as well as increased difficulty swallowing. Evaluation for an underlying malignancy included CT scans of the chest, abdomen, and pelvis, mammogram, breast ultrasound and biopsy, pelvic examination with transvaginal ultrasound, colonoscopy, and positron emission tomography scan; no tumors were detected. Within 1.5 weeks of initiation of therapy, there was significant improvement in cutaneous and muscular disease, including the unilateral lower extremity edema (Fig 3).

## Discussion

Although facial, especially eyelid, edema can be commonly seen in patients with dermatomyositis, generalized edema or anasarca is rare.^2^, ^3^, ^4^ Furthermore, it is even rarer for patients to present with unilateral extremity swelling, with only 6 previously reported cases in the internal medicine literature (Table I).^3^^,^^5^, ^6^, ^7^ For 5 of these patients, the unilateral limb edema developed as more widespread edema resolved, or it represented the initial site of edema and was followed by edema in additional sites.

**Table I:** Six previously reported cases of dermatomyositis with unilateral extremity swelling during the course of their disease^3^^,^^5^, ^6^, ^7^

Author	Age, sex	Case details	Myositis-specific antibody	Treatment
Chai et al, 2011^3^	62, M	Proximal muscle weakness Gottron papules Generalized swelling initially improved, and then the patient developed RUE swelling; subsequently had LUE swelling (same side as port-a-cath, but evaluation revealed no DVT or significant venous narrowing) Dysphagia	Unknown	Prednisone, MTX, IVIG
Chai et al, 2011^3^	23, F	Proximal muscle weakness Severe generalized rash Pitting edema of upper and lower extremities bilaterally initially improved, then developed RUE swelling (same side as port-a-cath, but evaluation revealed no DVT or significant venous narrowing)	Unknown	Prednisone, MTX, IVIG
Gorelik et al, 2001^5^	31, M	Muscle weakness and pain Maculopapular rash on the face, neck, hands, thighs, and feet Subsequent development of severe subcutaneous edema, began in the LUE and then spread to the limbs and trunk over 24 h Dysphagia	Unknown	Hydrocortisone, IVIG
Gorelik et al, 2001^5^	63, M	Proximal muscle weakness and pain Maculopapular rash on the face, neck, chest, and arms Subsequent development of severe edema and pain of the left forearm	Unknown	Sodium bicarbonate
Mukkera et al, 2021^6^	56, F	Proximal muscle weakness Urticarial rash on the face, arms, hands, and upper back Bilateral upper extremity and subsequently LLE pitting edema Dysphagia	NXP-2 (PM-SCL positive)	Prednisone, HCQ, MTX, AZA, IVIG
Werner de Castro et al, 2006^7^	40, M	Proximal muscle weakness Heliotrope and truncal and extremity rash Subsequent development of swelling in the RUE, then the RLE (imaging showed a DVT of the RUE); the patient went on to develop anasarca	Unknown	Prednisone, MTX, AZA, IVIG

*AZA*, Azathioprine; *DVT*, deep vein thrombosis; *HCQ*, hydroxychloroquine; *IVIG*, intravenous immunoglobulin; *LLE*, left lower extremity; *LUE*, left upper extremity; *MTX*, methotrexate; *RLE*, right lower extremity; *RUE*, right upper extremity.

Of the 6 patients with dermatomyositis outlined in Table I, only 1 reported with a myositis antibody profile and it was NXP2-positive.^6^ In 2017, Albayda et al^1^ demonstrated in a cohort of 56 patients with dermatomyositis with anti-NXP2 antibodies that there was a statistically significant increase in the likelihood of subcutaneous edema when compared to patients with NXP2-negative dermatomyositis (36% vs 19%; *P* = .01).^1^ In addition, patients with subcutaneous edema had more significant proximal and distal muscle weakness.^1^ Furthermore, it has been shown that the presence of anti-NXP2 antibodies was associated with significant dysphagia (74% vs 39%, *P* = .006).^8^ Though the underlying etiology is unclear, it has been hypothesized that edema in patients with dermatomyositis could be due to excessive vascular permeability, coexisting vasculitis, or inflammation of muscle tissue.^5^ In general, the presence of edema in patients with dermatomyositis has been associated with heightened disease severity, warranting timely and aggressive treatment.^9^

Because our patient with NXP-2-positive dermatomyositis had persistent unilateral lower extremity edema accompanied by erythema, she was initially diagnosed as having cellulitis, then based upon CT results, panniculitis was considered in the differential diagnosis, and due to histologic findings, erythema multiforme was also added. Diseases later in their course are often easier to diagnose, and the greater the number of previous discordant diagnoses, the greater the suspicion of an alternate diagnosis. The lesson is that the edema seen in patients with dermatomyositis can be localized, albeit in unusual locations, as well as generalized.
